# Visual versus Automated Evaluation of Chest Computed Tomography for the Presence of Chronic Obstructive Pulmonary Disease

**DOI:** 10.1371/journal.pone.0042227

**Published:** 2012-07-27

**Authors:** Onno M. Mets, Ewoud J. Smit, Firdaus A. A. Mohamed Hoesein, Hester A. Gietema, Reinoud P. H. Bokkers, Mohamed Attrach, Saskia van Amelsvoort-van de Vorst, Ernst Th Scholten, Constantinus F. M. Buckens, Matthijs Oudkerk, Jan-Willem J. Lammers, Mathias Prokop, Pim A. de Jong

**Affiliations:** 1 Department of Radiology, University Medical Center Utrecht, Utrecht, The Netherlands; 2 Julius Center for Health Sciences and Primary Care, University Medical Center Utrecht, Utrecht, The Netherlands; 3 Department of Radiology, University Medical Center Groningen, Groningen, The Netherlands; 4 Department of Pulmonology, University Medical Center Utrecht, Utrecht, The Netherlands; 5 Department of Radiology, Radboud University Nijmegen Medical Centre, Nijmegen, The Netherlands; University of Navarra, Spain

## Abstract

**Background:**

Incidental CT findings may provide an opportunity for early detection of chronic obstructive pulmonary disease (COPD), which may prove important in CT-based lung cancer screening setting. We aimed to determine the diagnostic performance of human observers to visually evaluate COPD presence on CT images, in comparison to automated evaluation using quantitative CT measures.

**Methods:**

This study was approved by the Dutch Ministry of Health and the institutional review board. All participants provided written informed consent. We studied 266 heavy smokers enrolled in a lung cancer screening trial. All subjects underwent volumetric inspiratory and expiratory chest computed tomography (CT). Pulmonary function testing was used as the reference standard for COPD. We evaluated the diagnostic performance of eight observers and one automated model based on quantitative CT measures.

**Results:**

The prevalence of COPD in the study population was 44% (118/266), of whom 62% (73/118) had mild disease. The diagnostic accuracy was 74.1% in the automated evaluation, and ranged between 58.3% and 74.3% for the visual evaluation of CT images. The positive predictive value was 74.3% in the automated evaluation, and ranged between 52.9% and 74.7% for the visual evaluation. Interobserver variation was substantial, even within the subgroup of experienced observers. Agreement within observers yielded kappa values between 0.28 and 0.68, regardless of the level of expertise. The agreement between the observers and the automated CT model showed kappa values of 0.12–0.35.

**Conclusions:**

Visual evaluation of COPD presence on chest CT images provides at best modest accuracy and is associated with substantial interobserver variation. Automated evaluation of COPD subjects using quantitative CT measures appears superior to visual evaluation by human observers.

## Introduction

Emphysema and airways disease are common incidental findings on computed tomography (CT) performed for other reasons, offering the potential to identify subjects with undetected chronic obstructive pulmonary disease (COPD) [1]. COPD is one of the leading causes of death [2], [3], and is expected to account for one in every 25 deaths in the developed world [2]. The disease is predominantly caused by tobacco exposure and is characterized by chronic airflow obstruction caused by emphysema and airways disease [4]. Since early smoking cessation prevents COPD disease progression [5], [6] and evidence suggests that early intervention improves outcome [7], [8], early diagnosis is crucial in managing this disease [9], [10]. Unfortunately, symptoms occur late in course of the disease and early stages are substantially underdiagnosed [11], [12]. Additionally, COPD is a predictor of cardiovascular mortality [13] and lung cancer [14], [15]. Given these facts, and given that chest imaging is among the most commonly ordered radiological examinations, often ordered by non-pulmonary specialists in patients with an unknown COPD status, there has been considerable interest in the use of chest imaging to identify subjects with COPD. However, the general conclusion is that conventional chest radiography is insensitive in identifying mild to moderate COPD-related abnormalities [16]–[19]. Contrarily, COPD-related abnormalities (ie. airways disease and emphysema) are probably more readily detectable on chest CT as compared to conventional radiography. The Lung Screening Study supports this superior accuracy by showing that chest CT depicted 2.5 times more COPD-related changes compared to chest radiography [20].

Recently, it has been reported that using an automated CT model based on quantitative measures of emphysema and air trapping, identification of COPD subjects in a lung cancer screening setting was feasible with reasonable accuracy [21]. However, the reliability and accuracy of human observers to visually evaluate COPD presence on CT images is unknown. Therefore, we aimed to determine the diagnostic performance of human observers with various levels of expertise to visually evaluate COPD presence on CT images, and compare this to the performance of automated evaluation based on quantitative CT measures.

## Methods

### Ethics statement

This study was performed within the setting of the population-based Dutch Belgian Lung Cancer Screening Trial (NELSON-trial; ISRCTN63545820) [22], which was approved by the Dutch Ministry of Health and by the local ethical review board (‘Medisch Ethische Toetsingcommissie University Medical Center Utrecht’). To study COPD, expiratory CT acquisition was added to the screening protocol (ie. inspiratory CT and pulmonary function testing) in our center, starting July 2007. This addition was separately approved by the local ethical review board of the University Medical Center Utrecht (approval 03-040/C). Written informed consent was obtained from each participant.

### Study population

**Figure 1:**
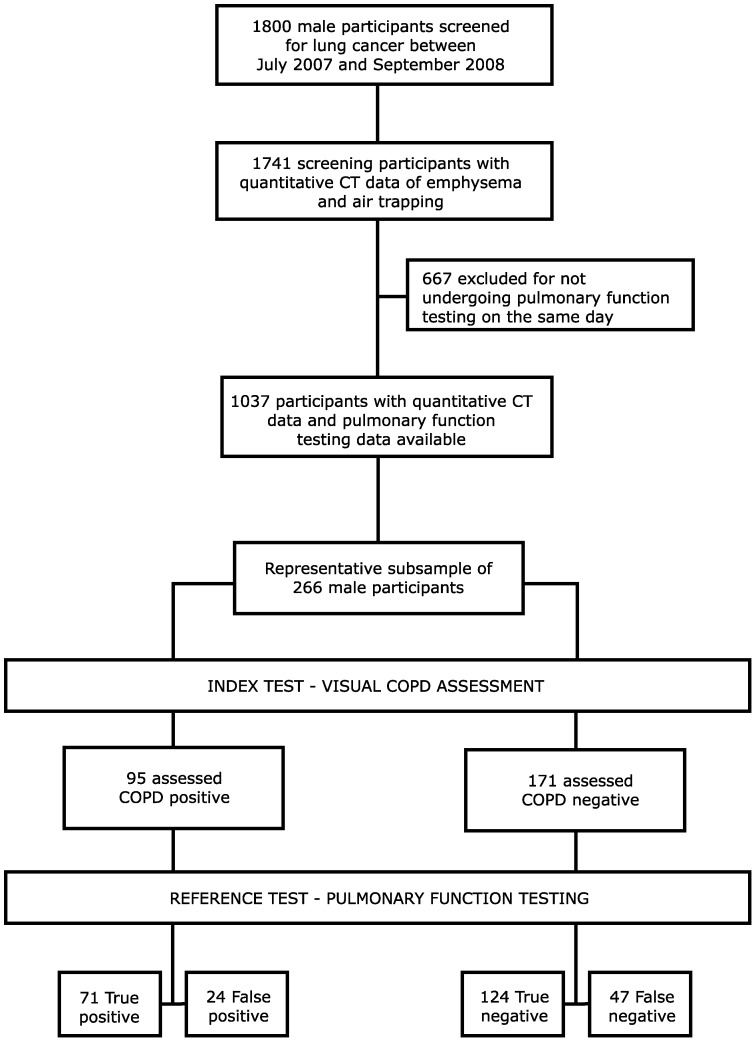
Flow diagram of the study. Flow diagram showing the selection of the study population from the total screening trial cohort. The index test presented is for the observer with the highest positive predictive value.

The NELSON-trial enables valuable research into the early stages of COPD, which is more difficult in clinical routine because early COPD is not an indication for chest CT in our routine practice. Participants were all current or former heavy smokers meeting the inclusion criteria of the screening trial, as described previously [22]. Briefly, participants were heavy smokers between the ages of 50 and 75 year with at least 16.5 packyears of smoking history who were also physically fit enough to undergo potential surgery. For the present study we included a random sample of 266 male individuals who had lung function testing and a paired inspiratory and expiratory CT scan obtained on the same day between July 2007 and September 2008. This cohort is a representative sample of the total screening population. The comparison between our study population and the total screening trial population is shown in Table 1. A flow diagram of the study is shown in Figure 1.

**Table 1:** Comparison between the included subsample of participants and the total cohort of screening participants in the study period between July 2007–September 2008.

		Study population^a^	All screening participants^b^
		(N = 266)	(N = 1,741)
Age in years			
	Mean ± SD	62.5±5.0	62.6±5.4
CT emphysema (%)			
	Mean ± SD	1.74±3.34	1.67±3.01
	Median [P25–P75]	0.78 [0.40–1.58]	0.76 [0.39–1.49]
CT air trapping (%)			
	Mean ± SD	84.4±5.9	84.1±6.5
	Median [P25–P75]	84.9 [80.9–88.4]	84.7 [80.2–88.3]

Comparison using Mann-Whitney U test revealed no significant differences between the two groups.

a data in the randomly selected subsample of male participants;

b data in the total group of male participants screened in the study period

### CT scanning

Volumetric CT in inspiration and at end-expiration was obtained from lung bases to lung apices after standardized breathing instructions by a trained radiographer. CT images were acquired with 16×0.75 mm collimation (Brilliance 16P; Philips Medical Systems, USA), and images with slice thickness of 1.0 mm at 0.7 mm increment were reconstructed using a smooth kernel (B-filter; Philips). Dose settings were adjusted to body weight: subjects weighing 80 kg or less received 120 kVp at 30 mAs for the inspiratory acquisition and 90 kVp at 20 mAs for the expiratory acquisition. Subjects weighing over 80 kg received 140 kVp at 30 mAs for the inspiratory acquisition and 120 kVp at 20 mAs for the expiratory acquisition.

### Pulmonary function testing

Pulmonary function testing without bronchodilator administration was performed on the same day as CT imaging. Spirometry was performed with ZAN equipment (ZAN Messgeräte GmbH, Oberthulba, Germany), according to the American Thoracic Society and European Respiratory Society guidelines [23]. The lung function testing included forced expiratory volume in the first second (FEV_1_) and the ratio of FEV_1_ over forced vital capacity (FEV_1_/FVC). The reference standard for COPD was a FEV_1_/FVC ratio less 0.70 [4].

### Visual evaluation of CT images

Eight observers with various levels of expertise in evaluating chest CT images [24] participated in this study. The observers were one specialized chest radiologist (P.A.J), one senior radiologist (E.Th.S), one senior-resident in radiology with chest radiology specialty (H.A.G), two junior residents in radiology (R.P.H.B, M.A), one clinical research coordinator evaluating lung cancer screening chest CT images (S.A.V), and two MDs performing COPD research (F.M.H, O.M.M); see Table 2 for more detailed information.

**Table 2:** Expertise levels and experience of the human observers.

	Job Title	Expertise level^a^	Reading chest CT^b^ (years)
Observer 1	MD researcher	I	0
Observer 2	MD researcher	I	2
Observer 3	Junior resident	II	2
Observer 4	Junior resident	II	2
Observer 5	Clinical research coordinator	II	7
Observer 6	Senior resident	IV	8
Observer 7	Senior radiologist	V	34
Observer 8	Chest radiologist	V	10

a level of expertise based as on Reference [24]: *I* has knowledge and some skills, *II* acts under full supervision, *III* acts under limited supervision, *IV* acts without supervision, *V* supervises and teaches;

b Years since the observer started reading and evaluating chest CT scans; Observer 1 to 5 were considered ‘less experienced observers’ and observer 6 to 8 were considered ‘experienced observers’.

All CT images were anonymized and presented to the observers in a randomized order on a 3D research workstation (iXviewer, Image Sciences Institute, Utrecht, The Netherlands). For each case, the paired inspiratory and expiratory CT scans were shown alongside each other. The observers were able to view each scan completely and in any direction, corresponding to clinical routine. The observers were asked to judge whether lung function impairment was present in the case presented (ie. COPD present or absent), based on their evaluation of the presence and extent of emphysema, air trapping, airway abnormalities or any other finding on CT imaging. They were also provided with some basic patient characteristics, similar as applied in the automated evaluation (ie. age, body mass index, smoking status and smoking history; see next paragraph). To closely resemble daily practice, visual evaluation of the cases was performed without a prior consensus meeting. Each of the 266 cases was evaluated by all eight observers. To study intraobserver agreement, a subset of 30 random cases was evaluated a second time by all observers.

### Automated evaluation of CT images

COPD presence was automatically evaluated, using a CT model that includes quantitative measures of CT emphysema and CT air trapping, age, body mass index, smoking status and smoking history. The model has previously been described in detail elsewhere [21]. In summary, the predicted probability for COPD presence was calculated using a regression equation (Probability_COPD_ = −11.400+0.9036*CT emphysema(log)+0.1519*CT air trapping −0.0645*BMI+0.0083*packyears (−0.7115 if former smoker). Based on the calculated probability, subjects were dichotomized as either COPD subjects or non-COPD subjects according to an optimal cut-off value [21].

### Statistical Analyses

Kappa (κ) values were calculated in order to assess intraobserver and interobserver agreement. Agreement was classified as poor when κ was 0.20 or less, fair when between 0.21 and 0.40, moderate when between 0.41 and 0.60, good when between 0.61 and 0.80, and very good when higher than 0.80 [25]. Both the automated and the visual evaluation were compared to the reference standard of pulmonary function testing, and diagnostic performance was calculated in terms of the sensitivity, specificity, positive predictive value (PPV), negative predictive value (NPV) and accuracy, all with 95% confidence intervals. Results are presented separately for the less experienced and experienced observers.

Diagnostic performance was compared between each observer and the automated evaluation by the CT model [26].

All analyses were performed with SPSS Version 15.0 for Windows (SPSS, Chicago, Illinois, USA). A p-value below 0.05 was considered statistically significant.

## Results

### Study population

Our study population consisted of 266 heavily smoking male subjects with a mean ± standard deviation age of 62.5±5.0 years. Detailed study population characteristics are presented in Table 3.

**Table 3:** Characteristics of the 266 study participants.

Characteristic		Values
Age, years [mean ± SD]		62.5±5.0
BMI, kg/m^2^ [mean ± SD]		26.8±3.4
Smoking status		
	Current smoker [n (%)]	135 (50.8)
	Former smoker [n (%)]	131 (49.2)
Packyears, median [P25–P75]		38 [28–46]
FEV_1_, % predicted [mean ± SD]		93.6±17.0
FEV_1_/FVC, % [mean ± SD]		69.3±9.2
Airflow limitation [n (%)]^a^		118 (44.4)
	Mild obstruction [n (%)]	73 (27.4)
	Moderate obstruction [n (%)]	40 (15.0)
	Severe obstruction [n (%)]	5 (1.9)

a airflow limitation was defined as FEV_1_/FVC ratio less than 70% and classified as mild (FEV_1_≥80%), moderate (50%≤FEV_1_<80%) and sever (FEV_1_<50%);

SD = Standard deviation; FEV_1_ = forced expiratory volume in the first second; FEV_1_/FVC = ratio of FEV_1_ over forced vital capacity

### Observer agreement in CT-based evaluation of COPD presence

The intraobserver agreement ranged from a κ-value of 0.28 to 0.68 (median 0.64) for the less experienced observers, and from 0.49 to 0.53 (median 0.49) for the experienced observers. The interobserver agreement for the less experienced observers yielded κ-values between 0.18 and 0.55 (median 0.36). The interobserver agreement for the experienced observers yielded κ-values between 0.35 and 0.57 (median 0.40).

The agreement between each less experienced observer and the automated CT model yielded κ values between 0.12 and 0.30 (median 0.28). For the experienced observers this ranged between 0.20 and 0.35 (median 0.33). Results on the observer agreement are listed in Table 4.

**Table 4:** Intra- and interobserver agreement for CT based identification of COPD.

	Observer 1							
Observer 1	0.64	Observer 2						
Observer 2	0.39	0.28	Observer 3					
Observer 3	0.49	0.32	0.68	Observer 4				
Observer 4	0.25	0.18	0.38	0.68	Observer 5			
Observer 5	0.46	0.28	0.55	0.33	0.53	Observer 6		
Observer 6	0.55	0.35	0.36	0.32	0.48	0.49	Observer 7	
Observer 7	0.53	0.31	0.48	0.43	0.55	0.57	0.49	Observer 8
Observer 8	0.30	0.35	0.32	0.25	0.35	0.35	0.40	0.53
Automated CT Model	0.30	0.12	0.28	0.23	0.30	0.20	0.33	0.35

Data given are Kappa (κ) values.

### Diagnostic performance for CT-based evaluation of COPD presence

In our study population, 44.4% (118/266) of the subjects had COPD according to the reference standard. The percentage of subjects with suspected COPD after visual evaluation of the CT images by the human observers ranged from 25.9% to 60.2%. The accuracy of the less experienced observers ranged from 58.3% to 62.4%, and the positive predictive value ranged from 52.9% to 60.9%. For the experienced observers this was 64.7% to 73.3% for the accuracy, and 64.6% to 74.7% for the positive predicted value.

The percentage of subjects with suspected COPD after automated evaluation by the CT model was 38.0%. The automated CT model had an accuracy of 74.1% and a positive predicted value of 74.3%. Table 5 specifies the diagnostic performance measures for each observer and for the automated CT model.

**Table 5:** Diagnostic performance measures with 95% confidence interval for CT-based evaluation of COPD presence.

	Sensitivity	Specificity	PPV	NPV	Accuracy
Observer 1	35.6	81.8	60.9	61.4	61.3
	(29.8–41.4)	(77.1–86.4)	(55.0–66.7)	(55.6–67.3)	(55.4–67.1)
Observer 2	54.2	61.5	52.9	62.8	58.3
	(48.3–60.2)	(55.6–67.3)	(46.9–58.9)	(57.0–68.6)	(52.3–64.2)
Observer 3	51.7	70.9	58.7	64.8	62.4
	(45.7–57.7)	(65.5–76.4)	(52.7–64.6)	(59.1–70.6)	(56.6–68.2)
Observer 4	73.7	50.7	54.4	70.8	60.9
	(68.4–79.0)	(44.7–56.7)	(48.4–60.4)	(65.3–76.2)	(55.0–66.8)
Observer 5	49.2	70.9	57.4	63.6	61.3
	(43.1–55.2)	(65.5–76.4)	(51.5–63.4)	(57.9–69.4)	(55.4–67.1)
Median Obs 1–5	51.7	70.9	57.4	63.6	61.3
	(45.7–57.7)	(65.5–76.4)	(51.5–63.4)	(57.9–69.4)	(55.4–67.1)
Observer 6	44.9	80.4	64.6	64.7	64.7
	(38.9–50.9)	(75.6–85.2)	(58.9–70.4)	(58.9–70.4)	(58.9–70.4)
Observer 7	50.8	78.4	65.2	66.7	66.2
	(44.8–56.9)	(73.4–83.3)	(59.5–70.9)	(61.0–72.3)	(60.5–71.9)
Observer 8	60.2	83.8	74.7	72.5	73.3
	(54.3–66.1)	(79.4–88.2)	(69.5–80.0)	(67.2–77.9)	(68.0–78.6)
Median Obs 6–8	50.8	80.4	65.2	66.7	66.2
	(44.8–56.9)	(75.6–85.2)	(59.5–70.9)	(61.0–72.3)	(60.5–71.9)
Automated CT Model	63.6	82.4	74.3	73.9	74.1
	(57.8–69.3)	(77.9–87.0)	(69.0–79.5)	(68.7–79.2)	(68.8–79.3)

Data given are percentages.

*PPV* positive predicted value; *NPV* negative predictive value

Comparison between the automated evaluation by the CT model and the visual evaluation by the human observers shows that all but two observers had a significantly worse diagnostic performance in either sensitivity or specificity, or both (0.001<p<0.05). Only the specialized chest radiologist clearly approached the diagnostic performance of the CT model (p = 0.79), while a clear trend was seen for the other, less experienced observer (p = 0.06).

## Discussion

In this study we report the diagnostic performance of human observers in identifying subjects with COPD using visual evaluation of lung cancer screening chest CT scans. Their performance was compared to the performance of automated evaluation of CT images. Accuracy of visual evaluation for COPD presence was modest, and the accuracy of the automated evaluation was higher than that of the observers. Diagnostic performance of the human observers seems to improve slightly with level of expertise, and approaches that of the automated model for the specialized chest radiologist. Nevertheless, intraobserver and interobserver variation was substantial, even in the most experienced observers. Our study demonstrates that although CT images contain diagnostic information related to COPD in a population with mainly early stages of disease, the reliability and diagnostic accuracy of visual evaluation is limited and certainly not better than automated evaluation.

The fairly low accuracy of visual evaluation for COPD presence shows that human observers experience difficulty in judging which lung abnormalities are functionally relevant. In addition, the limited intraobserver and interobserver agreement found indicates that human observers have their own subjective and inconsequent understanding of what COPD would look like on CT (ie. what type of abnormalities, and to what extent, will result in airflow obstruction and abnormal lung function). This finding is in line with previous literature that has shown that visual evaluation of emphysema, air trapping and airway wall thickening are prone to considerable interobserver variability [27]–[31]. This, together with the modest diagnostic accuracy, has clinical implications: the extensive and increasing use of CT imaging [32], combined with the commendable practice of radiologists to report all imaging findings, including the incidental and unrequested ones, may lead to an increase in subjects who are wrongfully stigmatized based on the presence of COPD-related abnormalities on CT. Consequently, our study urges radiologist to remain cautious in interpreting these abnormalities and in reporting previously unknown disease. Whenever COPD is suspected based on CT findings, confirmatory lung function testing is required and should always be suggested.

Since CT-based lung cancer screening in heavy smokers is now recommended in the US [33], [34] the chances to detect early COPD in high-risk subjects using screening CT images are increasing. At this stage, better understanding of functionally relevant CT abnormalities and improvement of observer agreement should be sought, which may lead to improved accuracy. On the other hand, identification of COPD can be based on automated evaluation using quantitative CT analysis, which we believe will become more important than that of visual evaluation; it is fast and inexpensive and the basic CT model, which at this stage includes only simple lung density measures and few patient characteristics, already performs better than the human observers. Its performance is approached only by the specialized chest radiologist, and it is unlikely that in daily practice the large amount of lung cancer screening CT scans will be reviewed by a specialized chest radiologist. Nevertheless, the quantitative approach needs to be further validated and improved, and clinical use might require more standardized CT operating procedures to limit differences between CT scanners and differences in breath hold procedures.

Our study is of importance since it addresses a common clinical problem, related to a disease with major healthcare impact. The main strengths are that we have used a representative sample of CT readers with various levels of expertise, and closely resembled clinical practice with 3D inspiratory and expiratory CT data and some clinical information. Also, we were able to provide data on a substantial number of subjects with early stages of COPD who are difficult to obtain in routine practice.

Our study has limitations. Firstly, spirometry was performed without administration of a bronchodilator, which is recommended to exclude asthma. However, we believe it is unlikely that this has significantly influenced the results because the prevalence of asthma in men between the ages of 50 and 75 years is only approximately 2% in the general population of the Netherlands [35], and our study population comprised only heavy smokers. Secondly, our study was limited to male subjects. This may limit the generalizability of our findings. Thirdly, our study evaluated functionally relevant lung abnormalities at the time of imaging. Given the cross-sectional nature of our study we cannot comment on whether observers identified subclinical abnormalities that may lead to abnormal lung function in the future. Lastly, we were unable to include more than one or two observers at each level of expertise, which impedes analysis within a group of similar experienced observers. Nevertheless, our results are based on a fairly large group of observers subdivided into a less experienced and experienced subgroup.

In conclusion, this study reports modest diagnostic accuracy of human observers in the visual evaluation for COPD presence on volumetric inspiratory and expiratory CT images in heavy smokers. Moreover, visual evaluation for COPD presence is associated with substantial observer variation. Our findings suggest that visual evaluation of CT scans for COPD presence is of limited diagnostic value, while there may be a role for automated evaluation. This may be important for the additional identification of COPD subjects in a CT-based lung cancer screening setting.

## References

[1] Lee CI, Forman HP (2010) What we can and cannot see coming. Radiology 257 (2) 313–314. 20959544 10.1148/radiol.10101437

[2] Mathers CD, Loncar D (2006) Projections of global mortality and burden of disease from 2002 to 2030. PLoS Med 3 (11) e442. 17132052 10.1371/journal.pmed.0030442 PMC1664601

[3] Murray CJ, Lopez AD (1997) Alternative projections of mortality and disability by cause 1990–2020: Global Burden of Disease Study. Lancet 349 (9064) 1498–1504. 9167458 10.1016/S0140-6736(96)07492-2

[4] Rabe KF, Hurd S, Anzueto A, Barnes PJ, Buist SA, et al (2007) Global strategy for the diagnosis, management, and prevention of chronic obstructive pulmonary disease: GOLD executive summary. Am J Respir Crit Care Med 176 (6) 532–555. 17507545 10.1164/rccm.200703-456SO

[5] Scanlon PD, Connett JE, Waller LA, Altose MD, Bailey WC, et al (2000) Smoking cessation and lung function in mild-to-moderate chronic obstructive pulmonary disease. The Lung Health Study. Am J Respir Crit Care Med 161: 381–390. 10673175 10.1164/ajrccm.161.2.9901044

[6] Anthonisen NR, Connett JE, Kiley JP, Altose MD, Bailey WC, et al (1994) Effects of smoking intervention and the use of an inhaled anticholinergic bronchodilator on the rate of decline of FEV1. The Lung Health Study. JAMA 272 (19) 1497–1505. 7966841

[7] Decramer M, Cooper CB (2010) Treatment of COPD: the sooner the better? Thorax 65 (9) 837–841. 20805184 10.1136/thx.2009.133355

[8] Godtfredsen NS, Lam TH, Hansel TT, Leon ME, Gray N, et al (2008) COPD-related morbidity and mortality after smoking cessation: status of the evidence. Eur Respir J 32 (4) 844–853. 18827152 10.1183/09031936.00160007

[9] Barnes PJ (2007) Chronic obstructive pulmonary disease: a growing but neglected global epidemic. PLoS Med 4 (5) e112. 17503959 10.1371/journal.pmed.0040112 PMC1865560

[10] Fiore MC, Baker TB (2011) Clinical practice. Treating smokers in the health care setting. N Engl J Med 365 (13) 1222–1231. 21991895 10.1056/NEJMcp1101512 PMC4494734

[11] Soriano JB, Zielinski J, Price D (2009) Screening for and early detection of chronic obstructive pulmonary disease. Lancet 374 (9691) 721–732. 19716965 10.1016/S0140-6736(09)61290-3

[12] Bednarek M, Maciejewski J, Wozniak M, Kuca P, Zielinski J (2008) Prevalence, severity and underdiagnosis of COPD in the primary care setting. Thorax 63 (5) 402–407. 18234906 10.1136/thx.2007.085456

[13] Hole DJ, Watt GC, Davey-Smith G, Hart CL, Gillis CR, et al (1996) Impaired lung function and mortality risk in men and women: findings from the Renfrew and Paisley prospective population study. BMJ 313 (7059) 711–715. 8819439 10.1136/bmj.313.7059.711 PMC2352103

[14] Brenner DR, McLaughlin JR, Hung RJ (2011) Previous lung diseases and lung cancer risk: a systematic review and meta-analysis. PLoS One 6 (3) e17479. 21483846 10.1371/journal.pone.0017479 PMC3069026

[15] Sekine Y, Katsura H, Koh E, Hiroshima K, Fujisawa T (2011) Early detection of COPD is important for lung cancer surveillance. Eur Respir J 39 (5) 1230–40. 22088970 10.1183/09031936.00126011

[16] Woodring JH, Phillips BA, West JW, Ulmer J, Cooper JK (1991) A prospective evaluation of plain radiographic signs of chronic obstructive pulmonary disease. J Thorac Imaging 6 (2) 14–21. 10.1097/00005382-199104000-00005 1856897

[17] Fraser RG (1974) The radiologist and obstructive airway disease. Caldwell Lecture, 1973. Am J Roentgenol Radium Ther Nucl Med 120 (4) 737–775. 10.2214/ajr.120.4.737 4595267

[18] Takasugi JE, Godwin JD (1998) Radiology of chronic obstructive pulmonary disease. Radiol Clin North Am 36 (1) 29–55. 9465867 10.1016/s0033-8389(05)70006-3

[19] Shaker SB, Dirksen A, Bach KS, Mortensen J (2007) Imaging in chronic obstructive pulmonary disease. COPD 4 (2) 143–161. 17530508 10.1080/15412550701341277

[20] Pinsky P, Freedman M, Oken M, Kvale P, Caporaso N, et al (2007) Prevalence of non-cancer-related abnormalities on low-dose spiral computer tomography versus chest radiograph in a screening population. Thorax 62 (2) 190. 10.1136/thx.2006.073288 PMC2111253 17287309

[21] Mets OM, Buckens CF, Zanen P, Isgum I, van Ginneken B, et al (2011) Identification of chronic obstructive pulmonary disease in lung cancer screening computed tomographic scans. JAMA 306 (16) 1775–1781. 22028353 10.1001/jama.2011.1531

[22] van Iersel CA, de Koning HJ, Draisma G, Mali WP, Scholten ET, et al (2007) Risk-based selection from the general population in a screening trial: selection criteria, recruitment and power for the Dutch-Belgian randomised lung cancer multi-slice CT screening trial (NELSON). Int J Cancer 120 (4) 868–874. 17131307 10.1002/ijc.22134

[23] Miller MR, Hankinson J, Brusasco V, Burgos F, Casaburi R, et al (2005) Standardisation of spirometry. Eur Respir J 26 (2) 319–338. 16055882 10.1183/09031936.05.00034805

[24] ten Cate O, Snell L, Carraccio C (2010) Medical competence: the interplay between individual ability and the health care environment. Med Teach 32 (8) 669–675. 20662579 10.3109/0142159X.2010.500897

[25] Brennan P, Silman A (1992) Statistical methods for assessing observer variability in clinical measures. BMJ 304 (6840) 1491–1494. 1611375 10.1136/bmj.304.6840.1491 PMC1882212

[26] Hawass NE (1997) Comparing the sensitivities and specificities of two diagnostic procedures performed on the same group of patients. Br J Radiol 70 (832) 360–366. 9166071 10.1259/bjr.70.832.9166071

[27] Washko GR (2010) Diagnostic imaging in COPD. Semin Respir Crit Care Med 31 (3) 276–285. 20496297 10.1055/s-0030-1254068 PMC4334134

[28] Cavigli E, Camiciottoli G, Diciotti S, Orlandi I, Spinelli C, et al (2009) Whole-lung densitometry versus visual assessment of emphysema. Eur Radiol 19 (7) 1686–1692. 19224221 10.1007/s00330-009-1320-y

[29] Malinen A, Erkinjuntti-Pekkanen R, Partanen K, Rytkonen H, Vanninen R (2002) Reproducibility of scoring emphysema by HRCT. Acta Radiol 43 (1) 54–59. 11972463 10.1080/028418502127347448

[30] Mascalchi M, Diciotti S, Sverzellati N, Camiciottoli G, Ciccotosto C, et al (2011) Low agreement of visual rating for detailed quantification of pulmonary emphysema in whole-lung CT. Acta Radiol 53 (1) 53–60. 22114019 10.1258/ar.2011.110419

[31] Barr CC, Berkowitz EA, Bigazzi F, Bode F, Bon J, et al (2012) A Combined Pulmonary-Radiology Workshop for Visual Evaluation of COPD: Study Design, Chest CT Findings and Concordance with Quantitative Evaluation. COPD (2) 151–9. 22429093 10.3109/15412555.2012.654923 PMC3752926

[32] Brenner DJ, Hall EJ (2007) Computed tomography–an increasing source of radiation exposure. N Engl J Med 357 (22) 2277–2284. 18046031 10.1056/NEJMra072149

[33] American Lung Association (2012) Providing Guidance for Lung Cancer Screening: The American Lung Association Interim Report on Lung Cancer Screening. Available: http://www.lung.org/finding-cures/research-news/new-screening-guidelines/lung-cancer-screening.pdf. Accessed 2012 May.

[34] Wood DE, Eapen GA, Ettinger DS, Hou L, Jackman D, et al (2012) Lung cancer screening. J Natl Compr Canc Netw 10 (2) 240–265. 22308518 10.6004/jnccn.2012.0022 PMC6467530

[35] Nationaal Kompas Volksgezondheid [National Public Health Compass] Available: http://www.nationaalkompas.nl/gezondheid-en-ziekte/ziekten-en-aandoeningen/ademhalingswegen/astma/omvang. Accessed 2012 May.

